# Editorial: The early detection of neurodegenerative diseases: an aging perspective

**DOI:** 10.3389/fnagi.2025.1765664

**Published:** 2026-01-12

**Authors:** Paolo Abondio, Mirco Masi, Shaoyu Wang

**Affiliations:** 1 IRCCS Institute of Neurological Sciences of Bologna (ISNB), Bologna, Italy; 2 Computational and Chemical Biology, Italian Institute of Technology (IIT), Genoa, Italy; 3 School of Dentistry and Medical Sciences, Charles Sturt University, Orange, NSW, Australia

**Keywords:** aging neuroscience, artificial intelligence, digital phenotyping, early detection, electrophysiology, longitudinal assessment, multimodal biomarkers, network analysis

Neurodegenerative disorders represent major challenges to modern medicine as they affect an increasing number of individuals as populations age (Jiang et al., 2025; Masi et al., 2023). Despite diagnostic advances, these diseases are typically identified only after substantial neuronal loss has occurred (Shabani and Hassan, 2023; Golde, 2009; Li et al., 2025). The earliest stages of these diseases such as Alzheimer's disease (AD), Parkinson's disease (PD) or vascular cognitive impairment (VCI) are gradual and often masked by compensatory mechanisms that sustain normal functioning (Masi et al., 2022; Gao et al., 2025; Hou et al., 2019; Scribano Parada et al., 2025). Therefore, early detection requires identifying the earliest measurable deviations from healthy aging—whether molecular, physiological, cognitive, or behavioral—before irreversible damage develops (Sperling et al., 2011; Wang and Antonova, 2019). This Research Topic “*The Early Detection of Neurodegenerative Diseases: An Aging Perspective*” brings together 10 original contributions that collectively expand the concept of “early” in neurodegeneration. Rather than treating aging as a passive risk factor, these studies reframe it as an active process that modulates vulnerability and resilience across biological systems (Abbatecola et al., 2024). Figure 1 synthesizes this view, outlining a multimodal framework that links molecular, physiological, cognitive, and functional domains through shared analytical tools and aging-related modifiers.

**Figure 1:**
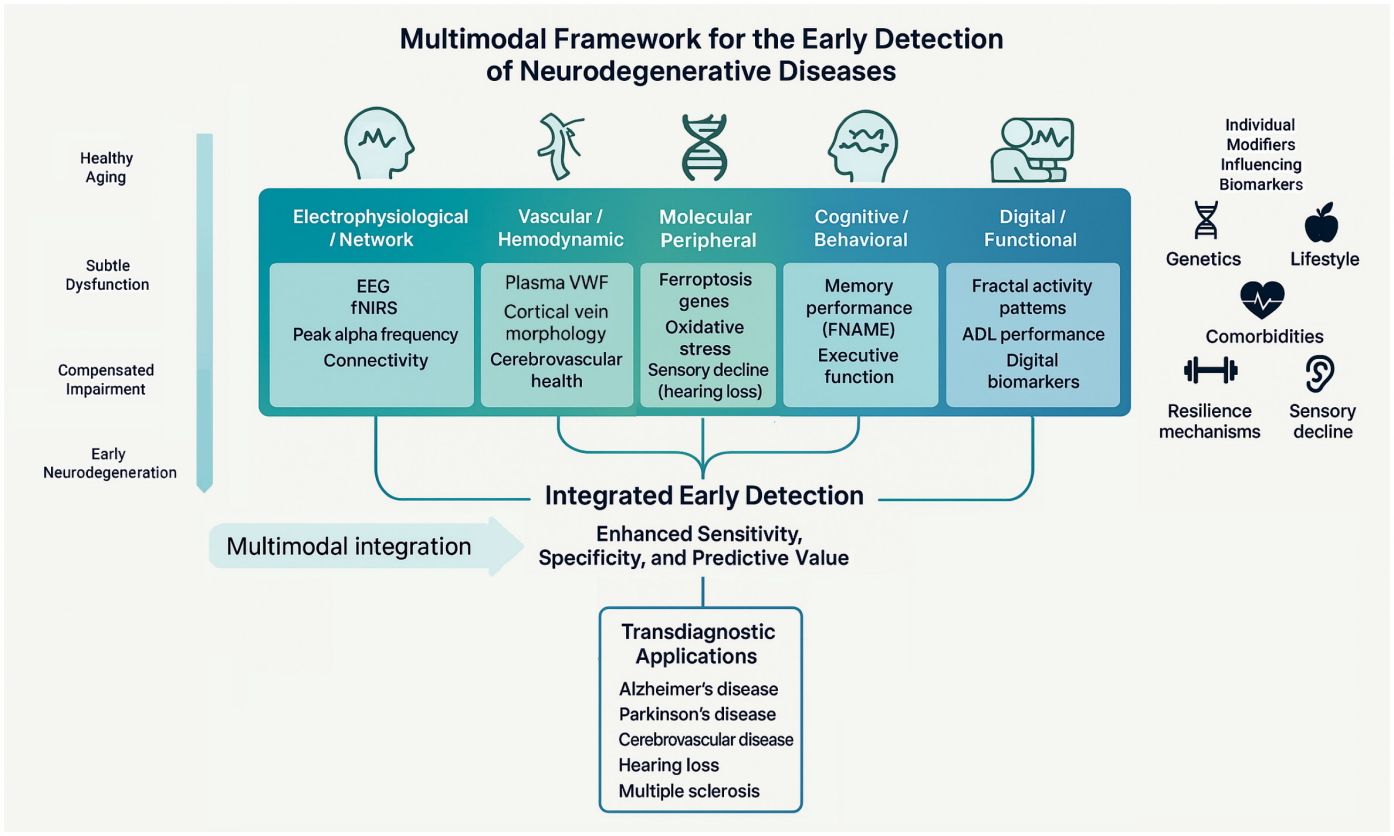
Multimodal framework for the early detection of neurodegenerative diseases. The diagram illustrates how diverse biomarker domains converge to support early detection across the aging-neurodegeneration continuum. Five complementary domains—electrophysiological/network (EEG, fNIRS connectivity), vascular/hemodynamic (VWF, cortical vein morphology, cerebrovascular health), molecular/peripheral (ferroptosis-related genes, oxidative stress, hearing loss), cognitive/behavioral (memory performance, executive function, depression networks), and digital/functional (fractal activity patterns, ADL performance, digital biomarkers)—collectively contribute to an integrated early-detection framework. Individual modifiers such as genetics, lifestyle, comorbidities, resilience mechanisms, and sensory decline modulate biomarker expression and interpretability. The integration of these multimodal signals enhances sensitivity, specificity, and predictive power, enabling transdiagnostic applications across Alzheimer's disease, Parkinson's disease, cerebrovascular disease, hearing loss, and multiple sclerosis (ADL, activities of daily living; EEG, electroencephalography; FNAME, face–name associative memory exam; fNIRS, functional near-infrared spectroscopy; VWF, von Willebrand Factor).

## From localized lesions to network-level dysregulation

A consistent theme across several contributions is the transition from regional lesion-based models to system-level interpretations of neural dysfunction. Paitel et al. systematically reviewed more than 120 electroencephalographic (EEG) studies in AD and mild cognitive impairment (MCI), highlighting reproducible patterns of reduced alpha-band connectivity, revealing that disrupted communication precedes overt neurodegeneration and supporting the view of AD as a disconnection syndrome. Zhao Y. et al. demonstrated that peak alpha frequency is a sensitive and robust electrophysiological marker of post-stroke cognitive impairment (PSCI), linking oscillatory slowing to cognitive decline across cortical regions and supporting strong associations with Montreal Cognitive Assessment (MoCA) scores. Together, these studies position EEG as an accessible, temporally precise window into early disconnection processes not visible to conventional structural imaging, as also supported by the leading editor Wang's research group work. This network perspective extends also to PD. Zhao J. et al. employed functional near-infrared spectroscopy (fNIRS) to reveal distinct cortical activation patterns during executive tasks. PD patients with MCI displayed enhanced interhemispheric connectivity, suggesting compensatory recruitment, whereas those with dementia showed network hypoactivation, consistent with structural atrophy and network collapse. Hence, electrophysiological and hemodynamic connectivity measures emerge as accessible tools capable of identifying transitional phases between normal and pathological aging, thresholding between adaptive and maladaptive reorganization and offering accessible functional biomarkers for longitudinal monitoring.

## Vascular contributions to cognitive decline within the neurodegenerative continuum

Vascular mechanisms emerge as another unifying element across this Research Topic. Fu and Hu reported that lower plasma von Willebrand Factor (VWF) levels predict faster longitudinal cognitive decline and greater hippocampal and entorhinal cortex atrophy in older adults without dementia, underscoring the prognostic significance of vascular biomarkers in apparently healthy individuals, capturing subclinical vulnerabilities that precede faster deterioration. Xie et al. used susceptibility-weighted imaging and artificial intelligence (AI)–based segmentation to quantify superficial cortical veins, revealing correlations between venous morphology, tau levels, and cognitive performance, linking cerebrovascular architecture to neurodegenerative proteinopathy. Together, these contributions suggest neurovascular integrity as both biomarker and mechanistic driver within the neurodegenerative continuum, linking microvascular dysfunction, neuroinflammation and early neurodegenerative cascades.

## Molecular and peripheral correlates of neural vulnerability

Early biomarkers can also be detected outside the central nervous system. Yuan et al. identified ferroptosis-related gene signatures as diagnostic and therapeutic targets in hearing loss, an adult-onset peripheral disorder with mechanistic parallels to neurodegeneration. Genes such as *MEF2C* and *NEDD4*, implicated in age-related auditory nerve deterioration, also appear in deafness associated datasets, suggesting potential cross-organ biomarkers of oxidative stress and cell death. Their multi-step approach—combining transcriptomics screening, machine-learning model selection, and experimental validation—highlights how integrated pipelines can accelerate the discovery of peripheral indicators of neural vulnerability. Sensory decline may thus serve as a sentinel of neurodegenerative risk, reflecting broader early neurobiological vulnerability.

## Behavioral, cognitive, and computational indicators of early vulnerability

The identification of sensitive cognitive and behavioral indicators remains essential to early detection. Liu et al. validated the modified Face–Name Associative Memory Exam (mFNAME) as a reliable tool for detecting subtle mnemonic deficits, showing robust associations between performance and network efficiency within the default mode and medial temporal systems, even in asymptomatic individuals. Zhào H. et al. used actigraphy to analyze fractal dynamics of physical activity in older adults with cerebral small vessel disease, demonstrating that reduced temporal complexity correlates with disease severity. Their findings highlight the value of digital phenotyping into early detection frameworks as a real-world complement to traditional cognitive assessments. Sun et al. applied network analysis to explore interactions between activities of daily living (ADL) limitations and depressive symptoms in older adults, identifying “toileting” as a bridging node between physical and affective domains. This work illustrates how behavioral interdependencies can reveal early, multidimensional vulnerabilities. Mohamed et al. showed that baseline cardiorespiratory fitness, but not aerobic capacity, predicts cognitive and motor outcomes in multiple sclerosis (MS), emphasizing disease heterogeneity and suggesting that resilience mechanisms may be domain-specific rather than generalized. Across these behavioral and computational approaches, AI, network modeling, and longitudinal analytics enhance detection sensitivity and help delineate complex patterns across datasets. As these tools grow more sophisticated, maintaining mechanistic interpretability alongside predictive accuracy remains essential.

## Toward a transdiagnostic framework for early detection

Collectively, these contributions support a multimodal, aging-informed framework for early detection, indicating neurodegeneration not as isolated diseases but as overlapping trajectories shaped by aging-related biological processes. Across conditions such as AD, PD, VCI, hearing loss, and MS, the early manifestations share overlapping mechanisms like oxidative stress, vascular dysregulation, disrupted connectivity, and impaired compensatory plasticity. Each study captures a different facet of the gradual erosion of neural adaptability. Figure 1 integrates these findings by positioning the aging trajectory against multiple observational levels. Analytical tools such as AI-based modeling, network approaches, and multimodal integration provide the connective framework, while aging-related factors (genetics, lifestyle, vascular health, resilience mechanisms and sensory decline) modulate vulnerability. Future research should build on this integrative model, combining biomarkers across scales to predict the transition from resilience to vulnerability. Ultimately, early detection should not only identify disease risk but also map the biological processes that maintain or erode neural adaptability across the lifespan. From this perspective, aging is not a passive risk factor but an active landscape shaping neurodegenerative trajectories. The challenge ahead is to optimize and operationalize this framework to identify individuals at risk not by the presence of disease, but by the loss of resilience.
